# Supplementary material for: Are carbon emissions trading and green financial instruments synergistic? -Comprehensive quantitative research based on content analysis
Source: PLoS One. 2024 Mar 7;19(3):e0298601. doi: 10.1371/journal.pone.0298601 (PMC10919601; doi:10.1371/journal.pone.0298601)
Supplement: S1 Data — (ZIP) [file pone.0298601.s001.zip › Data/地级市Z_F工作报告.zip!/║■─╧/╔█╤⌠╩╨/└┤╫╘╣1⁄2╓┌║┼í╛┬φ┐╦╩2╛▌═°í┐_2019.docx]

各位代表：

现在，我代表市人民政府，向大会报告政府工作，请予审议，并请各位政协委员提出意见。

一、2018年工作回顾

一年来，我们坚持以习近平新时代中国特色社会主义思想为指导，全面贯彻落实党的十九大和十九届二中、三中全会精神，在省委、省政府和市委的坚强领导下，坚持稳中求进工作总基调，着力推进高质量发展，着力打好“三大攻坚战”，着力振兴实体经济，着力保障和改善民生，接续解决了一批长期想解决的难事，办成了一批补短板、强弱项的大事，干成了一批打基础、利长远的实事，做成了一批邵阳人民期盼已久的喜事，在艰难困苦中求进取，在下沉压力中求发展，咬紧预期目标奋力前行，总体完成了全年目标任务。

这一年，我们克难破困抓发展，顶住防化政府债务风险大量压减项目、固定资产投资严重下滑、环保治污成批转产关停企业等重重压力，密集调度、精准发力，经济增长先抑后扬，最终赶超全省平均水平。邵东县重返全省县域经济十强行列。三一湖汽年产值突破50亿元。

这一年，我们“背水一战”抓脱贫，实现23.77万贫困人口脱贫、432个贫困村出列，新邵、武冈、绥宁3个贫困县市可望成功摘帽，开创了邵阳减贫史上的新纪元。

这一年，我们倾心民生造福祉，财政民生支出占比创下79%的新高，省定12件重点民生实事、化解城区超大班额、农村危房改造、易地扶贫搬迁、自然村通公路、行政村通班车、村村建成标准化卫生室和农村人安饮水等任务圆满完成，解决人民群众上学难、看病难、住房难、出行难问题迈出历史性新步伐。气势恢宏的市体育中心建成投运，与已建成的市文化艺术中心形成“一江两岸、双璧矗立”的城市新地标，市级文化、体育设施跃居全省领先水平。

这一年，我们咬紧牙关干产业，彩虹特种玻璃、家用厨电智能制造、桑德再生资源循环利用等一批重大产业项目建成投产，省“五个100”工程首战告捷，彩虹集团、三一湖汽、柳桥国际3家企业及创新产品在省委经济工作会议上受到通报表彰，高质量发展点燃了星星之火。

这一年，我们重拳出击治污染，“散乱污”企业得到有效治理，饮用水水源地得到有效保护，蓝天、碧水、净土保卫战有效推进，中央环保督察和“回头看”、省环保督察交办问题有效整改，空气质量、水质量有效改善。

这一年，我们基础设施建设传捷报，怀邵衡铁路全线通车，邵阳人民为之期盼、为之奋斗几十年的梦想终于美梦成真。怀邵衡铁路建成通车，宣告湖南正式形成高铁省内闭合圈；怀邵衡铁路建成通车，结束了邵阳西部洞口、隆回无铁路的历史，洞口、隆回、邵阳县一步到位直接迈入“高铁时代”；怀邵衡铁路建成通车，联通了我国东西沪昆、南北京广两大高铁大动脉，标志着邵阳成为高铁交通枢纽，宣告邵阳全面进入“高铁时代”。武冈机场运营第一年，旅客吞吐量就突破46万人次，一步跃升为全省第5名。

这一年，我们创新发展奏凯歌，“一村一辅警”“邵阳快警”经验做法，作为新时代继承和发展“枫桥经验”的典型在全国推介、全省推广，成为全省叫得响、全国有影响的邵阳品牌。

这一年，我们创建“国字号”品牌传喜讯，恰逢改革开放四十周年，邵阳获批“湘南湘西承接产业转移示范区”，这是我市获得的第一个国家层面的最大平台，是我市历史上最大的发展机遇。上半年，我市东部城镇群规划获批，邵阳被省政府正式确立为省域副中心城市、湘中湘西经济文化中心。下半年，我市保税改革试点方案获省批准，邵阳保税仓正式投运。年底又传来喜讯，邵阳海关被国家正式批准。这些国、省重大平台，必将成为引领邵阳未来发展的强大引擎，推动邵阳高质量发展走出湖南、走出中国、走向世界。

一年来的工作成效主要体现在八个方面：

（一）经济运行稳中有进、进中提质

11项主要经济指标增速有9项高于或达到全省平均水平。其中，7项排全省前5位。尤其是体现高质量发展的结构性指标增速多处于全省前5位水平。

经济结构趋优。开始由高速增长阶段向高质量发展转变。投资结构上，沉着应对政府投资与控债碰头挤压，积极推进产业项目建设年“五个100”大行动，市委、市政府分两批组织党政代表团赴浙江学经验、换脑筋、招项目，到“广交会”开展专场推介，到“大湾区”、到“长三角”抓招商、引产业，带动工业投资、工业技改投资、高新技术产业投资逆势上扬，创下了近年来的投资新高。工业投资增长53.1%，高于全省20.7个百分点，排全省第1位。其中，工业技改投资增长72.2%，高于全省34.1个百分点，排全省第3位。高新技术投资增长43.5%。民间投资增长24.1%，成为拉动投资增长的主要动力。财税质量明显提升，地方收入中非税占比下降9.98个百分点，为近40年来最低。产业结构上，三次产业比由上年的19.7：35.4：44.9优化为16.1：34.9：49.0。经济结构上，全市最终消费支出对经济增长贡献率上升到85.8%。进出口总额增长31.7%，高于全省5.2个百分点，排全省第5位，是全省唯一一个连续六年保持20%以上增速的市州。外贸企业出口退（免）税总额17.54亿元，仅次于长沙，排全省第二位。

创新驱动提质。全社会研发经费投入同比增长141.2%，创历史新高。财政科技支出增长101.1%。净增国家高新技术企业47家，总数达到119家，同比增长65.27%。纳入统计口径的高新企业个数达到813家，比上年净增175家，增长27.43%。新增省重点实验室（工程技术研究中心）2个、获得省科技进步二等奖2项。全市发明专利申请量绩效评估排全省第四位。8家本土企业获得“创客中国”中小微企业创新创业大赛奖励，邵东智能制造技术研究院2个项目获评全国制造业“双创”平台试点示范，广信科技获评“湖南省工业质量标杆企业”。

放活市场增效。“放管服”改革唤醒市场主体活力，新登记市场主体41680户、增长14.2%，连续三年实现两位数增长。落实中央、省、市一系列优惠政策，进一步优化经济发展环境，减税降费为企业减少各类成本约30亿元。新增融资担保公司2家、增资30亿元，新增担保余额9.2亿元、增长76.1%。市本级“企业过桥专项资金”为企业提供偿债过桥资金2亿元，市中小企业信用担保公司为80户企业提供担保5亿元。规模工业企业实现利润90.39亿元，增长27.9%，高于全省10.3个百分点。持续推进普惠金融，隆回县被授予“湖南省首批普惠金融示范区”，邵东县、隆回县纳入“湖南省资本市场县域工程试点县”。

（二）“三大攻坚战”战绩不俗

积极有效防范化解政府性债务风险。高位推动，密集调度，令行禁止，坚守“三不”“三保”原则。全面摸清底数，锁定隐性债务存量，严格控制增量，编制切实有效化债方案。市本级“停、缓、调、撤”两批公益性基础设施项目，压减投资457.88亿元。通过争取置换债券额度、清理闲置土地、清收应收款项、加快政府性资产资源归集、处置、拍卖，压减政府公用经费等方式，积极筹集偿债资金，化解存量隐性债务34.14亿元，国家审计署公告的72.33亿元违规举债，已整改到位69.01亿元，平台公司整合转型有序推进。在风险面前，我们沉着应对，保持定力，精准施策，有效作为，产业建设没“踩空”，项目投资没“断崖”，“三保”资金没“爆雷”，平台公司没“塌方”，还本付息没“断链”。

用心用情用力推进精准扶贫。坚持以脱贫攻坚统揽经济社会发展全局，行业扶贫强势发力，“五个一批”精准落地，“脱贫攻坚作风建设年”活动卓有成效。市本级财政安排专项扶贫资金1.12亿元，各县市区整合涉农资金30.7亿元投入扶贫，完成帮扶项目998个，建成了一批产业基地和“扶贫车间”，组建了一批合作社，进驻了一批龙头企业，培养了一批种养大户，发展了一批乡村旅游，带动了87万贫困群众增收。新宁“崀山”脐橙入选中央电视台国家品牌计划—广告精准扶贫项目。隆回金银花、洞口油茶、新邵常春藤等产业项目列入全省20个贫困县特色产业扶贫创新试点。贫困户危房改造1.74万户，易地扶贫搬迁3.5万人，武冈市集中安置经验在全国推广。新增贫困劳动力转移就业14924人，绥宁县选聘农村劳务经纪人帮助贫困户解决就业的经验在中央电视台《新闻联播》栏目推介。发放教育扶贫资金1.58亿元，惠及16.42万贫困学生。健康扶贫“先诊疗后付费”“一站式”结算服务落实到位。生态扶贫安排生态护林员1万人。兜底保障1.23万户。贫困村电商扶贫、光伏扶贫、农网改造、安全饮水、标准化卫生室实现全覆盖。

打好污染防治攻坚战。持续推进中央和省环保督察反馈问题整改、办结问题整改上千个，持续推进环保基础设施补短板，持续强化建设工地和城市卫生管理，持续推动“散乱污”企业和老污染区治理，持续抓实“河长制”工作，治污工作取得明显进展。完成宝庆电厂发电机组超低排放改造等70个工业减排项目，完成百余家餐饮油烟改造；黏土砖厂全部退出，城区“黄标车”全部淘汰。2018年市区空气质量综合指数4.34，较上年下降14.4%，由上年的排名全省第13位上升到第9位。空气优良天数305天，比上年增加26天，空气优良率85%，较上年提高8.4%；重污染天气5天，比上年的18天减少了13天。全市11个省级工业园区污水处理厂全部建成并在线监测运行。关闭水源保护区内河道采砂场18个，关闭或搬迁各类企业35家，完成223个规模畜禽养殖场退养。完成饮用水水源一二级保护区内所有排污口截流，市区5条黑臭水体治理任务基本完成。全市6个国控断面、35个省控断面水质全部达到或优于3类标准，市区、各县城集中式饮用水水源和市级交界断面水质100%达标。邵东县铬污染农田土壤综合治理与生态修复全面完成，龙须塘老工业区重金属污染综合治理主体工程完工。

（三）产业建设卓有成效

产业建设来势喜人。省“五个100”工程进展顺利，6个省定重大产业项目完成投资35.85亿元，为年度计划的112%。三一湖汽环保智能自卸车和二纺机旋挖钻机创新产品按期投放市场，4个“500强”企业项目落地开建。彩虹特种玻璃项目点火投产，填补了我国特种玻璃空白，为打破国外垄断、打造“中国特种玻璃谷”打下了坚实基础，迈出了关键一步，为欠发达地区搞高科技产业项目树起了一个标杆、扬起了一面旗帜。全市211个重大产业项目累计完成投资289.3亿元。亚洲富士电梯、讯源电子、武冈百威啤酒等建成投产。新增“四上”单位751家，仅次于长沙，排全省第2位，连续5年居全省前3位。其中，新增规模以上工业企业255家，排全省第2位，连续5年居全省前3位。

重点项目推进有力。全市296个重点项目累计完成投资535.7亿元，120个项目实现全部投产或单项投产。19个省重点建设项目（含打捆项目）完成投资203亿元。邵阳电网完成投资13.2亿元，110千伏六岭变项目建成投产，结束了市中心城区无电源支撑的历史。潭邵高速公路（区段）大修工程全面完工。国家重点旅游项目“上堡古国”建设完成投资2.2亿元，黄桑4A级景区建设完成主体工程。

园区发展步伐加快。湘商产业园建设继续领跑全省，累计开工标准厂房501万㎡、竣工485万㎡，累计签约企业815家、入驻596家、投产512家，实现产值441.6亿元、税收18.6亿元，解决就业5.38万人。全市11个省级园区综合评价排名全省第6位，连续三年排位上升。邵东经开区推进“一区多园”建设，新打造了几个专业园区，工业智能化改造、产业转型升级斩获颇丰。邵阳发制品、邵东打火机、邵东箱包获批国家外贸转型升级基地。

（四）改革开放走深走实

供给侧结构性改革深入推进。以壮士断腕的决心关停退出“散乱污”企业35家，一批“僵尸”企业出清，一批流域区域污染企业关停搬迁。坚持“房住不炒”定位，房地产市场回归正常。多措并举降成本、补短板，为企业减税降费30亿元。全社会R&D经费投入占GDP的比重提高0.32个百分点。智能制造破题发展，来势喜人，一批企业走上高质量发展之路。

党和政府机关机构改革稳步推进。市级党委机关、政府机关和人大机关、政协机关机构改革全面启动、方案落地，群团改革总体完成序时任务，全市机构改革有条不紊顺利推进。

“放管服”改革取得新突破。进一步取消10项行政许可，下放11项行政权力事项，取消行政审批中介服务事项18项、证明材料64项，下放邵阳经开区232项经济管理权限全面落实，推进“多证合一、一照一码”登记制度改革和“四办”改革，完成2447项权力和290项公共服务事项目录梳理，116项压缩办结时限30%以上，500项做到“最多跑一次”。市本级“互联网+政务服务”一体化平台正式上线运行，与长沙等3个市率先与省平台对接。我市获评“全省发展开放型经济优秀市州”，新宁县获评“全省发展开放型经济优秀县”，三一湖汽获评“湖南开放强省十强外贸企业”。市公共资源交易中心获评全国公共资源交易行业“改革制度创新奖”。

保税改革成功试点。邵阳经开区公共保税仓和出口监管仓建成并获批运营，邵阳经开区连泰鞋业、隆回兴昂鞋业、洞口兴雄鞋业、邵东东亿电气建成自用型保税仓，保税改革成功迈出历史性的第一步。全市新增进出口实绩企业126家，总数达389家，其中148家实现业绩破零、43家实现倍增。2018年度湖南市州开放型经济引领能力报告出炉，邵阳排全省第六位。

农业农村改革取得新进展。农村土地确权登记颁证基本完成。全面启动农村集体产权制度改革，4152个集体经济组织完成清产核资。国有农场办社会职能改革扎实推进，完成确权发证94.52%。获评“第三次全国农业普查先进集体”。

南山国家公园体制试点取得新成效。《南山国家公园总体规划》已经省政府审定通过，整合修复碎片化、恢复保护原真性工作有序推进，试点区自然资源确权登记全面完成，资源状况“一张图”、登记“一个库”、管理“一张网”基本成型，并通过国家评估验收。《南山国家公园管理条例》已经省政府常务会议审议通过并提请省人大常委会审议。集体林权流转、生态移民搬迁、科研监测、产业退出、智慧管理等领域的工作全面启动，体制机制创新工作稳步推进。

（五）城市建管提质提档

“城市双修”全面启动。市体育中心投入运营，邵水西路、邵水东路、新城大道、湖口井路、紫霞路、邵檀路等周边路网基本完工，成为城区新景观。市城区首条隧道——新华路隧道全线贯通；新华南路二期和大祥路北段竣工通车；龙须塘路、学院路二期即将建成；白马大道、立新路二期加速推进；资江南岸防洪堤建设正式开工。气化邵阳一期建成投产，二期正式启动。资江两岸30万平方米饮用水水源地保护工程绿化全面完成，3万株树木进城新增绿地210公顷。

城市经营管理啃“硬骨头”。进一步规范招投标、招拍挂和项目评审、资金拨付等管理制度。有效处置闲置土地32宗、面积108.45公顷。落实土地储备“五统一”制度，坚持“熟地”“净地”出让，执行产业发展用地差别地价，依法规范土地出让底价设定，运用网拍网挂出让土地，市区经营性用地均价由2017年的139万元/亩上升到209万元/亩，最高出让单价突破312万元/亩。扎实推进市城区超期安置工作，努力破解超期安置和安置房过剩长期并存的困局。

扎实推进“三城同创”。对标对表开展创建活动，获得全国文明城市提名资格，在中央文明办年度暗访测评中获得较好成绩。《邵阳市国家森林城市建设总体规划》通过国家评审。“四边五年”绿色行动造林30.76万亩。洞口县获得“国家森林旅游示范县”称号。扎实推进国家健康城市创建，首获“全国无偿献血先进市”荣誉称号。新邵县成功创建省级卫生县城。

（六）乡村振兴开局良好

出台《乡村振兴战略实施意见》。突出产业兴旺。大力推进品牌强农行动。邵东廉桥镇被确定为全国“农业强镇”，洞口雪峰蜜橘产业基地获评省优质农副产品供应基地，绥宁“贵太太”茶油获评湖南“十大农业企业品牌”，邵阳县油茶被授予“国家气候标志”优质农产品称号。“百里脐橙连崀山，产业扶贫耀三湘”农旅融合大放异彩。全市农产品加工企业增加到5985家，休闲农业经营主体发展到2852个，农民专业合作社成长到3783家。致力生态宜居。稳步推进农村人居环境综合整治，隆回县率先启动重点镇污水处理设施建设。全市新建垃圾中转站186座，完成农村改厕3.9万户，拆除“空心房”282万平方米，拆旧复垦新增耕地5135亩。启动连片建房示范点16个，启动市级美丽乡村建设办点示范168个村。全面落实“河长制”，处置僵尸船894艘，数量居全省首位；“一河一警长”做法获全省推介。推动乡风文明。发挥“四会”等群众组织作用，有效遏制铺张浪费、大操大办、买码赌博、厚葬薄养等陈规陋习。深入开展社会主义核心价值观宣传教育，推进文化惠民活动下乡村。推进乡村治理。落实“四议两公开”“一事一议”。依法查处农村涉黑涉恶村霸和宗族恶势力、黑恶团伙案件。开展乡风文明村、最美乡贤评选和文明家庭创建活动。关爱农村留守儿童“代管家长制”获全国创新案例。

（七）竭尽全力为人民办实事、谋福祉

累计投入资金32亿元，平稳消除66人以上超大班额3868个，超额50%完成省定年内目标任务，超大班额比例从20.39%降至0.7%，取得历史性突破。市一中新校区完成主体工程建设，全市中小学D级危房全面消除。市中心医院东院投运。农村适龄妇女“两癌”免费检查和孕产妇免费产前筛查目标人群实现全覆盖。成功举办第十三届省运会成年组赛事，邵阳代表团分获成年组、青少年组团体总分第二名、第六名，为我市近20年来在省运会上取得的最好成绩。省第九届少数民族传统体育运动会获得团体总分第三名。新建和建成县级文体中心6家、村级文化中心1866个。邵阳县白仓镇、城步苗族自治县儒林镇、隆回县虎形山瑶族乡被评为2018—2020年“湖南民间文化艺术之乡”。新创作了《山乡工匠》《火种》《磨豆腐》《边桃边李》《儿大女大》等一批优秀剧目。城镇新增就业5.1万人，失业人员再就业2.76万人，失业率控制在4.5%以内。农民工工资治欠保支成效明显，为1.1万名农民工追回工资3440.84万元。改造城市棚户区22553户、国有工矿棚户区624户，分配公共租赁住房1.1万套，发放租赁补贴2.51万户、3035万元。发放个人住房公积金贷款23亿元。农村最低生活保障标准提高到每人每年3420元。食品药品安全形势稳定。

（八）社会治理有力有效

全力推进“公安消防大建设三年行动”，全市刑事发案下降15.52%，命案发案下降44.83%，“两抢”发案下降79.2%。“一村一辅警”推动农村社会和谐稳定，“邵阳快警”实现社会面管控能力明显增强。依法开展扫黑除恶专项斗争，开展“两打两控”和禁毒“百日会战”，持续加大涉黄、涉赌、涉毒、涉枪涉爆打击力度，全市治安形势进一步好转。扎实推进安全生产“春雷2018”专项整治、“打非治违百日行动”“强执法防事故”“秋季攻势”行动，全市生产安全事故起数和死亡人数分别下降34%和20.6%，学生溺亡人数下降57%，未发生重特大事故，安全生产形势总体平稳。深入开展“信访法治化建设年”、信访“三无”创建活动，年内创建信访“三无”乡镇（街道）55个、“三无”村（社区）1393个，绥宁县和洞口县实现“进京非访”零登记。

认真执行市人大及其常委会的决议决定，自觉接受市人大的法律监督、工作监督和市政协的民主监督，150件代表建议和268件委员提案全部办结。认真执行重大行政决策程序规定，切实做到依法行政。严格落实党风廉政建设要求，“三公经费”下降17%。审计监督持续发力，查出各类违规资金45亿元。

坚持党管武装，加强国防动员和后备力量建设，国防教育、双拥共建、民兵调整改革工作有效开展。与此同时，档案、民族、宗教、工商、质监、海事、人防、国安、双拥、国防动员、粮食、供销、气象、邮政、通讯、工会、共青团、妇女儿童、科协、残联、文联、工商联、红十字会、贸易促进会、台湾事务、外侨港澳事务、驻外办事机构、高等院校、科研院所等工作取得新的成绩。

成绩的取得，是党中央、国务院、省委、省政府和市委正确领导的结果，是全市人民团结奋斗的结果。在此，我代表市人民政府，向全市各族人民，向人大代表、政协委员，向各民主党派、工商联、无党派人士和人民团体，向驻邵部队、武警官兵，向所有关心、支持邵阳发展的朋友们，表示衷心的感谢和崇高的敬意！

事非经过不知难。在经济下沉压力加大、转型升级困难增多、外需受限与内需不足碰头、转型升级与技术封锁碰头、稳增长与防风险碰头的情况下，能够取得这些成绩，实属不易，其经验也弥足珍贵。突出的主要有三条：一是始终坚持正确的政治方向。坚持以习近平新时代中国特色社会主义思想为指导，推进中央、省委巡视整改、环保督察整改、脱贫攻坚作风建设，提高了广大干部的政治站位，树牢了“四个意识”、坚定了“两个维护”。做好巡视整改，进一步增强了贯彻落实习近平新时代中国特色社会主义思想的自觉性和坚定性，激励了干部担当作为。推进环保督察整改，倒逼各级干部树立正确的政绩观，为高质量发展打扫了战场。高标准推进脱贫攻坚，贫困群众得到更多实惠，促进了社会和谐。认真落实党风廉政建设责任制和意识形态责任制的要求，坚定不移反腐倡廉、从严治党，严格执行中央八项规定精神，着力营造良好政治生态、自然生态和社会生态，为高质量发展提供了坚强保障。二是始终坚持发展为第一要务。面对稳中有变、变中有忧的新情况，保持定力，从容应对，咬定目标不放松，坚持发展硬道理，坚持高质量发展，坚持推进供给侧结构性改革，坚定实施“创新引领开放崛起”战略，积极推进“二中心一枢纽”建设，积极开展产业项目建设年活动，坚定不移做好发展工作，坚持用发展解决前进中的困难和问题。三是始终坚持新发展理念和正确的政绩观。坚持向超越财力盲目修大马路、建大广场等形象工程说不，向以环境污染为代价的粗放式发展说不，向依靠土地财政和房地产经营城市的传统发展模式说不，推动树牢新发展理念，实现工作重心和发展行动向供给侧结构性改革、保障和改善民生、推进实体产业建设聚焦，向高质量发展转变，集中有限财力，更多投入民生领域和产业建设，为高质量发展打下好的基础。

在总结成绩的同时，我们也清醒地看到：投资增长乏力，经济下行压力增大，个别经济指标没有实现预期，个别县经济滑坡比较严重；经济发展环境欠优，综合营商成本仍然较高；新旧动能转换不快，传统产业转型升级缓慢，新兴产业支撑能力不强；实体经济面临困难较多，民营企业和中小微企业融资难、融资贵的问题尚未根本解决；“三大攻坚战”问题不少，安全生产、信访稳定、扫黑除恶、禁毒以及社会面管控等风险点较多；一些政府工作人员担当意识不强，主动作为欠缺，极少数的甚至违纪违规、违法犯罪，人民群众还不满意。对此，我们要直面问题，切实加以解决。

二、2019年工作安排

2019年是新中国成立70周年，是全面建成小康社会关键之年。做好2019年工作意义重大、至关重要，必须以习近平新时代中国特色社会主义思想为指导，全面贯彻党的十九大和十九届二中、三中全会精神，认真贯彻落实中央、省委经济工作会议精神和市委决策部署，坚持稳中求进工作总基调和“六稳”方针，坚持新发展理念，坚持推动高质量发展，坚持以供给侧结构性改革为主线，坚持市场化改革、扩大高水平开放，坚持创新引领开放崛起，继续打好“三大攻坚战”，全力推进承接产业转移示范区建设，放手发展实体产业特别是先进装备制造产业，放手发展民营经济，加快建设“二中心一枢纽”，保持定力、提振信心，攻坚克难、担当作为，努力保持经济持续健康发展和社会大局稳定，为全面建成小康社会收官打下决定性基础，以优异成绩庆祝中华人民共和国成立70周年。

全市经济社会发展主要预期目标是：地区生产总值增长7.5%-8%，规模工业增加值增长7.5%，固定资产投资增长10%，财政总收入增长6%以上，社会消费品零售总额增长10.5%，实际利用外资增长12%，实际利用省外境内资金增长15%，进出口总额增长15%，城镇居民人均可支配收入增长8.5%，农民人均可支配收入增长10%，居民消费价格涨幅保持在3%左右，城镇调查失业率控制在4.5%以内，万元GDP能耗下降3.5%。

围绕上述目标任务，重点抓好七个方面的工作：

（一）奋力推进产业兴邵，“承接产业转移示范区建设”干在实处、走在前列

抢先一步大抓产业承接。对内，要苦练内功，咬紧牙关改善基础设施、打造承接平台、优化营商环境、降低营商成本，特别是把海关平台、通关平台、保税平台建好，把人工智能、工业互联网、物联网等新型基础设施建起来，把“互联网＋政务服务”“最多跑一次”和配套服务搞到位。对外，要以“浙江精神”“狼道精神”走出去招商，以最优惠的政策、最优质的服务招项目，以最优势的资源、最优越的产业链引项目，用最优秀的干部、最优厚的待遇去抢项目、引人才。月月要有项目签约，季季要有项目开工，年年要有项目投产，一年下来，要实实在在干成一批大项目、好项目，尤其是“500强”和重大产业链项目。围绕彩虹特种玻璃上下游产业链招商，打造全产业链的“中国（邵阳）特种玻璃谷”，形成百亿产业链；围绕智能厨电、智能五金、智能机器人三大智能制造基地和邵东智能制造技术研究院产业孵化园招商，打造“浙江产业园”“广东产业园”“德国精品五金产业园”，形成几个百亿智能制造小园区；围绕三一湖汽、通达汽零、维克液压、纺织机械、讯源电子、东亿电气、东方神鹰等优势企业招商，打造几个百亿产业集群；围绕发制品、小五金、打火机、鞋帽、服饰箱包、体育用品等出口企业，招引高端合作伙伴，打造优质品牌，形成百亿出口基地。与此同时，鼓励扶持传统产业进行战略性升级改造，推动“设备换芯”“生产换线”“机器换人”，推动“邵阳制造”向“邵阳智造”转变、“邵阳产品”向“邵阳品牌”提升。

要用好一切优惠政策，采取一切有效措施，承接产业转移，主攻先进制造业，壮大实体产业。市、县两级筹集50亿元产业扶持引导专项资金、50亿元企业融资担保资金、5亿元引才引智专项资金，用于奖扶引进产业、科创和人才。发挥邵商优势，凝聚邵商力量，支持全球邵商抱团发起成立邵商银行。在承接产业转移、壮大实体产业、推动高质量发展宏伟征途中，哪怕“砸锅卖铁”，也要把产业项目干起来；哪怕遇到一千个困难，也要使出一万个招数、杀出一条血路来，是所谓“胸有雄兵百千万”“越是艰险越向前”！项目未着，绝不放手；不获成效，绝不罢兵！

精心打造“135”工程升级版。按照省委书记杜家毫同志的构想，适应产业招商、集群入驻、量身定制、个性服务、培育生态的新趋势新特点，发挥邵商优势，集聚邵商力量，加强政策支持，提供优质服务，营造良好环境，呼应承接产业转移，精心打造“135”工程升级版，在湘商产业园建设中再抢先一步、提升一级。

千方百计扩大有效投资。全年实施重点项目300个左右，年度投资400亿元左右；争取纳入省重点项目20个左右，年度投资200亿元左右。全力争取一批国省重大基础设施项目，加快推进呼南高铁邵永段、永新高速、崀山高速（白新高速）、呼北高速新化至武冈段、城龙高速前期工作，力争部分项目年内开工。加快推进邵阳火车南站建设，争取部分站房建成。实施重点产业项目200个，年度投资300亿元。

（二）强力推进改革开放，切实增强发展活力

走好改革再出发第一步。全面深化供给侧结构性改革。贯彻中央“巩固、增强、提升、畅通”八字方针。巩固“三去一降一补”成果，严格执行质量、环保、能耗、安全等法规标准，把“僵尸企业”、落后产能、风险库存、过度杠杆去掉，把超期安置、资产债务处置、融资平台整合转型、“三供一业”分离等遗留问题处理好。进一步落实减税、降费政策，坚决把要素成本、物流成本、制度性交易成本、综合营商成本降下来。加快产业建设、污染治理、脱贫攻坚以及重大基础设施等领域“补短板”。增强微观主体活力，规范政府行为，让各类市场主体都能公平参与竞争，促进正向激励和优胜劣汰，充分调动企业家的主观能动性，培育更多优质企业。提升产业链水平，支持实体产业、“互联网＋”和创新平台建设，培育和发展新兴业态和产业集群，不断增强经济后劲、提升发展品质。畅通国民经济循环，放宽市场准入，落实“非禁即入”，敞开生产、加工、流通、消费等各个环节，让本地市场充满活力，畅通经济“小循环”。积极参与国家“一带一路”建设，主动对接“长江经济带”和粤港澳大湾区，做好人才、制度、创新等服务对接，畅通经济“大循环”。推动城商行、农商行、农信社业务回归本源、服务实体，畅通经济“外循环”。着力推进“放管服”改革。简化行政审批，实行告知承诺制；明确审批标准和办理时限；加大审批权限下放园区力度，对园区申报事项“见章盖章”；加强中介组织监管，建立红、黑名单制度；推进“标准地”“区域评估”。落实《优化政务服务十条规定》，加快推进“四办”改革，建立“一口受理、全国通办”的审批“云”服务平台。收回市直部门所有的行政审批权和测绘评估权，撤销其相关机构，组建市行政审批服务局。将市政务服务中心的“实体大厅”与市政府网上审批的“虚拟大厅”深度融合，实现所有行政审批事项“一个网”办理和线上线下一个平台运行。统筹抓好市县两级政府机构改革。按照“一类事项原则上由一个部门统筹、一件事情原则上由一个部门负责”的要求推进政府机构改革，明确职能职责，简化办事程序，提高办事效率，降低政务成本，为企业法人和人民群众提供快捷、便利服务。各涉改单位要服从大局，抓紧完成转隶交接和“三定”工作，尽快进入角色、履职到位。持续深化农业农村改革。推进农村土地“三权分置”改革，深化农村集体产权制度改革。推进农村房屋不动产登记，盘活闲置农房和宅基地。

下好开放再扩大先手棋。深入实施“五大开放行动”，推进商贸流通载体建设“六个100工程”。加快推进保税物流中心、城南物流园、城东物流园、邵阳东（邵东）货场物流园、武冈保税仓工业物流园等大型商贸物流项目建设。争取湘欧快线延伸到邵阳。发挥邵商优势，做足东盟、北部湾文章，力争建立“邵阳——东盟”对接平台，推动“邵品出境”。加快推进邵阳公路口岸作业区和邵阳海关配套服务建设，积极申报邵阳铁路口岸，争取临时开放武冈机场航空货运口岸。全面推进县市“两仓”建设，认真做好邵阳B型保税物流中心前期工作，同步申报海关特殊监管区，建成邵阳进口商品保税展示交易中心。

抢占创新再提质制高点。培育发展高新技术企业，高新技术产业增长16%；力争发明专利申请量、拥有量和专利总量增长15%。大力支持“湖南特种玻璃研究院”建设。加强与中国科技开发院的战略合作，争取在邵阳设立孵化基地。启动市级农业星创天地认定，加快工业“云服务”平台、工业大数据中心等创新服务平台建设。落实高层次人才和紧缺人才的引进服务保障办法，让各类人才汇聚邵阳，让创新活力竞相奔涌。

（三）全力优化营商环境，放手发展民营经济

构建新型市场监管机制。实行宽进入严监管，全面推行“双随机、一公开”监管，强化信用监管，完善事中事后监管，除安全生产、环境保护事项之外，非经市主管领导批准对园区企业一律免检查、“零打扰”；因人为因素对园区企业实施水电气拉闸停供的，由断供部门赔偿企业全部损失。构建优惠政策落实机制。行政事业性收费全免，服务性收费由用事单位代缴，由此造成的经费短缺由同级财政予以保障。不折不扣做好减税降费和出口退税工作。争取省里对湘商产业园和重点工业项目土地“点供”政策，做到重点园区、重大项目用地“应保尽保”。创新融资担保机制，积极探索园区以股权投资吸引高新技术企业入园，由银行、保险、园区、企业联手互动，以反担保增信落实科创型企业和中小微企业融资免担保。对在邵创建（设立）国、省级研发中心的给予500万至5000万元奖励，对在邵创建的上市企业，一次性给予500万至1000万元奖励。构建亲清新型政商关系。建立政府、企业良性互动机制。在企业困难时，政府主动出招、援手；当企业安定后，政府主动放手、退出。落实领导干部联系帮扶企业制度，由第三方民调实施企业评议政府部门工作。严肃查处懒政怠政和“索拿卡要”，坚决铲除官商勾结和“三强”行为。

（四）努力推进问题整改，继续打好“三大攻坚战”

攻城拔寨决胜脱贫攻坚。吹响决战决胜总攻号，紧扣“两不愁三保障”目标和“六个精准”要求，使出“脱皮”干劲和绣花功夫，确保全市现存所有贫困县全部摘帽、所有贫困村全部退出、所有贫困人口全部脱贫，把戴在邵阳人民头上数千年的贫困“帽子”扔进太平洋去！加强驻村帮扶，做到精准精细。抓住产业扶贫、就业扶贫、行业扶贫、社会扶贫等关键环节，做好教育、医疗、住房、交通、饮水、收入等基本保障。加强扶贫资金监管，开展扶贫领域腐败和作风问题专项治理。进一步巩固脱贫成果，确保退得出、稳得住、不反弹、能致富。

持续推进污染防治。扎实开展“回头看”，严防“死灰复燃”。加大“散乱污”企业治理力度，坚决杜绝新上高污染高能耗项目。切实加强建筑工地、道路扬尘、工业粉尘、烟花爆竹、秸秆焚烧等重点领域管控，增加空气质量优良天数。持续推进资江流域锑超标问题整治和饮用水水源地保护区突出环境问题整改。大力推进“河长制”工作，着力开展河库清“四乱”专项整治行动，实现“样板河”创建乡镇全覆盖。有序开展受污地块治理与生态修复。

有力有效防化政府性债务风险。继续落实“停、缓、调、撤”，坚决遏制隐性债务增量。多方筹措化债资金，有效化解债务存量。做好债务展期降息工作，平滑债务风险。争取地方政府一般债券、化解大班额一般债券和土地储备、棚户区改造等专项债券额度，争取财政均衡性转移支付，确保在建项目不“烂尾”、续建项目不“断链”。加快推进平台公司实质性整合和市场化转型。加强和改进金融监管，持续打击非法集资、互联网金融犯罪活动。

（五）着力推进“一群一圈”建设，稳步实施乡村振兴战略

加快“一群一圈”建设。实施东部城镇群体系建设。以省批规划为依据，引导“四县三区”相向而行。加快主城区与东部地区各城关镇公共交通、主干电网、信息物流、服务平台等基础设施建设和产业园区、特色小镇联动发展，拉开东部城镇体系大框架。抓好已经在城镇稳定就业的农业转移人口落户工作，力争到2020年全市新增城镇人口100万，邵阳市本级建成“双百城市”。提升主城区建管品质。加快推进在建道路、断头路和微循环路网建设。推进资江南岸防洪堤项目建设，打造城市防洪闭合圈。力争桂花渡水厂扩能工程开工，推进犬木塘水库工程前期工作。大力实施电网建设三年行动计划，加快特高压主干电网和坚强智能电网建设，力争年内投资50亿元。推进气化邵阳二期建设。完善“大城管”执法体系，打好闲置土地清理、超期安置、“空心房”拆除和拆违控违攻坚战。加快充电桩运营系统建设，加紧公共停车场建设。推进城市智能交通管理系统建设，进一步消除交通堵点。精准对接全省特色产业小镇建设“422”专项，培育发展一批园区社区产区和谐共生、一二三产业融合发展且优势互补、各具特色的产业小镇和“明星”镇。重点打造邵东廉桥南国药都特色小镇，邵阳县下花桥蓝印花布特色小镇、五峰铺商贸和农产品加工特色小镇、隆回花瑶民俗特色小镇、新邵雀塘再生资源产业小镇等，坚决杜绝“圈地运动”，切实防止千镇一面、有镇无业。推进西部生态旅游圈建设。加快西部生态旅游圈建设总体规划编制，强化生态功能区保护，大力发展生态旅游、乡村旅游和休闲康养等美丽经济，变“种种砍砍”为“走走看看”，化绿水青山为“金山银山”。抓好南山国家公园体制试点。完善试点实施方案，力促国家正式批复；抓好公园详细规划编制、生态补偿、管理立法以及生态保护性基础设施建设，全面完成园区自然资源确权登记，扎实开展草山植被恢复、核心保护区生态移民搬迁和水电风电以及涉矿涉企产业有序退出，探索建立科普教育与生态旅游协调、资源保护与区域发展统一的国家公园建设新模式。加快国家森林城市创建。着力实施重点工程提质、建城区绿化和乡村绿化三大攻坚行动，加快补齐绿色通道、绿色水道、生态廊道等绿化短板。

抓好乡村振兴战略实施。积极实施“一县一品”战略，做大做强市区近郊蔬菜和苗木花卉、邵东中药材、新邵富硒农产品、邵阳县油茶、隆回金银花和龙牙百合、洞口雪峰蜜橘和茶叶、武冈卤制品、新宁崀山脐橙、城步乳业和有机蔬菜、绥宁南竹等优质农产品示范基地建设，推动百亿茶叶、百亿油茶产业发展。大力支持“邵阳红”“贵太太”、南山乳业等品牌建设和龙头企业发展。深入实施“互联网+现代农业”，做强电商平台。完善乡村便民服务和矛盾调解机制，提升基层社会治理能力。大力开展农村人居环境综合整治，切实做好垃圾、污水、厕所、空心房等重点领域治理。推广农村可再生资源回收处理“邵东模式”。采取“整县打包”方式，引进几家真正有实力的环保治理企业，力争开工几个垃圾焚烧发电项目，开建一批重点乡镇污水处理厂项目。

（六）尽力发展社会事业，切实保障和改善民生

优先发展教育事业。切实解决当前义务教育“城市挤、农村弱”、学前教育“入园难、入园贵”和学校教学“校内减负、校外增负”的突出问题。统筹推进城乡义务教育一体化改革，坚决完成化解城区大班额年度任务。完成湘中幼专后续建设和市一中新校区搬迁扫尾工程，力争十七中新校动工。加快实施“芙蓉学校”建设。大力发展学前教育，推进普惠性幼儿园建设。鼓励职业教育走向市场，推进校企、产教融合，提高技能人才培养质量。

全面推动文化繁荣。切实落实意识形态责任制职责。推进全国文明城市创建，加强社区、窗口等37个类别测评点位的标准化建设，推动政务环境、法治环境、市场环境等八大环境建设，开展形式多样的主题教育实践活动和各类整治行动，持续抓好文明村镇、文明单位、文明校园、文明家庭创建工作。加强基层公共文化设施建设，提升文化惠民工程效能。加快构建现代公共文化服务体系，深入开展群众文化活动，抓好地方戏曲传承发展，繁荣文艺创作。加快推进绥宁侗寨申遗。深入开展“扫黄打非”行动。加强网络治理，营造清朗空间。

加快建设健康邵阳。广泛开展全民健身活动。积极推动各县市区体育场馆和全民健身活动中心建设。启动实施基层医疗卫生机构能力提升、妇幼健康和健康促进、重点传染病防控、贫困人口大病和慢性病精准救治4个三年行动计划。加快推进市第二人民医院整体搬迁项目建设。加强人口监测管理，提升计生服务水平。不断增强食品药品监管力度和安全保障水平。

尽力办好民生实事。扎实办好省定重点民生实事。加强就业创业培训和职业技能培训，重点做好高校毕业生、农民工、退役军人等群体多渠道就业创业。扎实开展就业扶贫，推动农村贫困劳动力转移就业。妥善做好治理“散乱污”企业、化解过剩产能和处置“僵尸企业”过程中下岗职工再就业工作。继续提高城镇退休人员基本养老金和城乡居民基础养老金待遇水平。将大病保险报销比例提高到55%以上，年度补偿最高限额提高到30万元。加大超期安置工作力度，确保完成2954户超期安置任务。

（七）致力创新社会治理，确保社会大局安全稳定

加强社会风险防控。加强社会综合治理，严格落实矛盾纠纷排查预警和多元化解责任，切实做好社区矫正、安置帮教和禁毒、戒毒工作，加强困难群体法律援助。继续开展信访“三无”创建和“以村为主”活动，狠抓进京重复访越级访专项治理，不断提升信访工作法治化、规范化、科学化水平。

落实安全稳定保障。坚决落实安全生产党委政府领导责任、部门监管责任和企业主体责任，持续开展重点行业、重点领域安全专项治理，有效遏制较大事故，坚决杜绝重特大安全事故发生。开展“基层消防上台阶”三年行动，推进“两乡镇一交管中队”建设。建立健全社会稳定风险评估机制和应急管理机制，进一步发挥“一村一辅警”和“邵阳快警”在防范处置突发公共事件中的作用。持续开展扫黑除恶专项斗争。坚决完成省政府确定的5个县级强制隔离戒毒所建设，坚决打击制毒、贩毒犯罪，坚决遏制吸毒行为，坚决打赢禁毒人民战争。认真抓好防汛抗旱、森林防火、公共安全、食品药品安全等事关群众安危的事情，确保社会大局平安稳定。

加强国防动员和国防后备力量建设，支持国防和军队改革、建设，推进军民融合深度发展。全面贯彻党的民族政策和宗教工作基本方针，促进民族团结、宗教和睦。支持工会、共青团、妇联等人民团体更好发挥桥梁纽带作用。做好外事、侨务、港澳、对台工作。加强统计、史志、文史、档案、气象、地震、邮政等工作。

各位代表，时代是出卷人，我们是答卷人，人民是阅卷人。人民对美好生活的向往就是我们的奋斗目标，人民群众的获得感、幸福感、安全感就是政府工作的努力方向。我们要始终与人民心心相印，与人民同甘共苦，努力造就信念过硬、政治过硬、责任过硬、能力过硬、作风过硬的干部队伍，努力建设人民满意的政府。

加强政治建设。以政治建设为统领，旗帜鲜明讲政治，牢固树立“四个意识”，不断增强“四个自信”，坚决做到“两个维护”。认真贯彻落实党中央、国务院和省委、省政府的决策部署，在市委的领导下开展工作，讲政治、守规矩，确保政令畅通、令行禁止。

加强法治建设。把政府活动全面纳入法治轨道。依法接受市人大及其常委会的监督，坚持重大决策出台之前向市人大报告制度，自觉接受市政协的民主监督，主动接受社会和舆论监督。加强重大行政决策、规范性文件合法性审查，加强审计监督、统计监测，提高政府决策科学化、社会治理精准化水平。

加强作风建设。驰而不息纠“四风”，坚持不懈提效能。大兴调查研究之风，深入基层沾泥土、接地气、听真话、干实事，认真解决群众最盼、最急、最忧的问题，以公务人员的辛苦指数换取人民群众的幸福指数。落实“三个区分开来”，大兴干事创业之风，干出千帆竞发、百舸争流的生动局面。

加强廉政建设。认真落实全面从严治党主体责任和“一岗双责”，严格执行中央八项规定精神，严格控制“三公”经费和一般性支出，把有限的资源、财力用在发展产业、改善民生上来。聚焦公共权力、公共资源、公共资金、脱贫攻坚、工程招投标等重点领域，坚决斩断黑幕链条和利益输送，严肃查处不正之风和腐败问题，营造风清气正的政治生态和社会生态。

各位代表，做好今年工作责任重大、使命光荣，让我们更加紧密地团结在以习近平同志为核心的党中央周围，高举习近平新时代中国特色社会主义思想伟大旗帜，在市委的坚强领导下，齐心协力，埋头苦干，攻坚克难，奋发有为，为全面建成小康社会收官打下决定性基础，以优异成绩庆祝中华人民共和国成立70周年！
